# Charge-Ordering and Magnetic Transitions in Nanocrystalline Half-Doped Rare Earth Manganite Ho_0.5_Ca_0.5_MnO_3_

**DOI:** 10.3390/nano15030203

**Published:** 2025-01-27

**Authors:** Giuseppe Muscas, Francesco Congiu, Alessandra Geddo Lehmann, Giorgio Concas

**Affiliations:** Dipartimento di Fisica, Università di Cagliari, I-09042 Monserrato, CA, Italy; g.muscas@unica.it (G.M.); a.geddolehmann@gmail.com (A.G.L.); gconcas@unica.it (G.C.)

**Keywords:** canted antiferromagnet, charge ordering, nanostructured manganites, perovskites, spin-glass dynamics

## Abstract

This work investigates nanostructured Ho_0.5_Ca_0.5_MnO_3_, considered a model system of the Ln_0.5_Ca_0.5_MnO_3_ series of manganites with perovskite structures featuring small lanthanide (Ln) ions half-substituted by Ca ions. Here, we propose a modified hybrid sol–gel–solid-state approach to produce multiple samples with a single batch, obtaining very high crystalline quality and ensuring the same chemical composition, with an average particle size in the range 39–135 nm modulated on-demand by a controlled calcination process. Our findings evidence that, provided the crystalline structure is preserved, the charge-ordering transition can be observed even at the nanoscale. Additionally, this research explores the presence of glassy phenomena, which are commonly seen in this class of materials, to enhance our understanding beyond simplistic qualitative observations. Comprehensive characterization using DC and AC magnetometry, along with relaxation and aging measurements, reveals that the complex dynamics typical of glassy phenomena emerge only at the nanoscale and are not visible in the bulk counterpart. Nevertheless, the analysis confirms that even the sample with the smallest nanoparticles cannot be intrinsically classified as canonical spin glass.

## 1. Introduction

Perovskite manganites, exhibiting intriguing electronic and magnetic properties arising from the delicate interplay between charge, spin, orbital, and lattice degrees of freedom, continue to attract significant research interest due to their potential applications in various fields, including electronics, catalysis, and energy storage [1]. In particular, rare earth-doped perovskite manganite oxide nanostructures are gaining renewed vivid interest regarding their nanostructured form for their promising technological applications, ranging from magnetic memory devices, magnetic sensors, and spintronic devices to solid oxide fuel cells, magnetic refrigeration, and biomedicine [2]. Half-doped manganites of the series Ln_0.5_Ca_0.5_MnO_3_ (Ln = lanthanide) have been the subject of intense research due to the variety of interesting magnetic phenomena observed, such as charge ordering (CO), ferromagnetic (FM) and antiferromagnetic (AFM) ordering, and glass-like behavior [3,4,5]. These magnetic states are reported in the phase diagram of bulk compounds drawn as a function of the tolerance factor (*t*) [6]. This is defined as $t=\left(r_{A}+r_{O}\right)/\sqrt{2}\left(r_{Mn}+r_{O}\right)\;$ and calculated using the effective ionic radii in Ref. [7], where *r*_A_ is the average radius of Ln^3+^ and Ca^2+^, *r*_O_ the radius of O^2−^, and *r*_Mn_ the average radius of Mn^3+^ and Mn^4+^. The parent compounds have an orthorhombic perovskite structure (space group *Pnma*) for lanthanide ions from La to Tb (large ions) [8] and a hexagonal structure (space group *P6_3_cm*) from Dy to Lu (small ions) [9]. The half substitution of Ln^3+^ with the larger Ca^2+^ stabilizes the perovskite structure for all compounds [4]. The parent compound LnMnO_3_ contains only the trivalent Mn^3+^ ion. With the half substitution of the trivalent Ln^3+^ ion with the divalent Ca^2+^ ion, the manganese assumes the Mn^4+^ configuration for half of the ions and maintains the Mn^3+^ configuration for the other half. This creates the necessary condition for the onset of the charge-ordering phenomenon. This phenomenon, characterized by the spatial modulation of charge density within the crystal lattice, plays a pivotal role in dictating the electronic and magnetic behavior of these materials [10]. Originating from the competition between Coulomb interactions, kinetic energy, and lattice distortions, charge ordering manifests as the formation of alternating regions of higher and lower charge densities [11]. This periodic arrangement not only can give rise to exotic electronic phases, such as insulating stripes or charge-ordered domains, but also profoundly influences magnetic properties, including magnetic ordering, spin dynamics, and magneto-transport phenomena [6].

As for magnetism, the CO transition marks the passage between two paramagnetic states at a certain temperature (*T*_CO_). In the first state above *T*_CO_, Mn^3+^ and Mn^4+^ ions respond individually to the applied magnetic field [12]. As for the second state below *T*_CO_, a model must be considered of a state in which Mn-O-Mn pairs plus an electron are formed, called Zener polarons [12]. The charge-ordering transition has been observed in bulk compounds of the series around 250–300 K [4,6,13,14]. Instead, the suppression of the CO transition was observed in nanostructured compounds with large ions (La, Nd, Pr, and Sm) [15,16,17,18]. With a reduction in particles to nanometric dimensions, the ferromagnetic interactions between Mn ions (double Zener exchange) [19] are strengthened compared to the antiferromagnetic ones (Anderson super-exchange) [20], leading to the suppression of the CO transition.

At a lower temperature, a transition toward an AFM ordered state has been observed in several compounds of the series, both in bulk and nanocrystalline form, [4,6,21,22], evidenced also by neutron diffraction in the Ho compound [13]. In nanocrystalline compounds with Nd, Pr, and Sm, an FM order appears, coexisting with the AFM order [16,17]. In the lowest-temperature region, below about 45 K, the typical behavior of a glassy state (such as spin glass or cluster glass) characterized by magnetic frustration has been observed in bulk compounds. At nanometric dimensions, a glassy state also appears in compounds with Pr and Sm [17,18].

In this context, this work aims to investigate potential nanoscale effects on the magnetic properties of Ho_0.5_Ca_0.5_MnO_3_, selected as a model system of the Ln_0.5_Ca_0.5_MnO_3_ series with small Ln ions, thereby addressing a significant gap in the literature on the class of half-substituted manganites. The primary objective is to verify the suppression of charge ordering, often attributed to nanoscale finite-size effects, using a series of samples prepared from the same batch that differ solely in particle size. This approach eliminates potential structural and compositional differences between bulk and nanoscale samples that could arise from variations in preparation conditions. Furthermore, this research explores the presence of the relaxation phenomenon observed in this class of materials [4].

## 2. Materials and Methods

Ho_0.5_Ca_0.5_MnO_3_ nanoparticles were prepared using a sol–gel self-combustion process followed by calcination [23]. First, 2.4 mmol of Ho(NO_3_)_3_ 6H_2_O, (purity 99.9%), 2.4 mmol of Ca(NO_3_)_2_ 4H_2_O (purity > 97%), and 4.8 mmol of Mn(NO_3_)_2_ 4H_2_O (purity 98%) were mixed in a beaker containing 3 mL of deionized water and 4.5 mL of diethylene glycol (DEG, purity 99%). All precursors were procured from Alfa Aesar (Haverhill, MA, USA). The solution was kept at 80 °C under mechanical agitation on a hot plate for about 20 min to dissolve the nitrates. Then, the solution was heated to 170 °C until the formation of a dry gel after approximately 30 min. At this stage, the temperature was rapidly increased to 300 °C, inducing self-combustion with small flames, which was completed in a few minutes. The sample was collected from the beaker in the form of a dry powder and transferred to a crucible. Finally, the sample was divided into several parts and individually calcinated in a tube furnace (Carbolite Gero, Derbyshire, UK) at different temperatures to induce optimal crystallization and to control the growth of nanoparticles of different sizes. Powders were not pelletized to limit sintering and intergrain diffusion. For all the samples, the temperature was increased and then decreased at a rate of 5 °C/min. In the following, we will discuss the results for five samples calcinated at temperatures of 650, 750, 900, 1000, and 1100 °C. The samples are called “Tx”, where “x” is the calcination temperature.

X-ray powder diffraction patterns were collected using a Siemens (Munich, Germany) θ-2θ D5000 diffractometer (Cu Kα) equipped with a secondary-beam graphite monochromator for Kβ elimination. Data were collected for 20° ≤ 2θ ≤ 65° and refined using the Rietveld method by means of the software MAUD (version 2.9993) [24]. The analysis aims at minimizing the difference between the detected and simulated powder diffraction patterns using the reliability index parameters R_wp_ (weighted residual error), R_B_ (Bragg factor), and R_exp_ (expected R factor), from which the goodness of the fit *Gof = R_wp_/R_exp,_ = χ* is calculated [25,26]. The residual stress was determined by the analysis method implemented in MAUD, using the size–strain isotropic model. The analysis is developed based on the work of S. Matthies [27,28], an advanced theory based on an elastic model, which includes the effects of lattice preferred orientations, and it can refine more complex stresses than the pure axial case.

Transmission electron microscopy (TEM) analysis was performed using a Jeol JEM 1400 Plus microscope (Akishima, Tokyo, Japan) operating at 120 kV. The sample powders were ultrasonicated in octane, and a drop of the solution was dried on a carbon-coated copper grid for observation. The recorded images were analyzed with the software FiJI (version 2.15), a distribution of ImageJ (version 2) [29], manually measuring the minimal and maximal diameters of individual particles and their agglomerates. For the smaller samples, the limited thickness allowed the electron beam to define the size and shape of a significant number of particles. A log-normal function was fitted to the size distribution:

(1) P=AD w 2πexp−ln2xxC2w2

where *A* is the area of the peak, *w* is the standard deviation of the natural logarithm of the variable *x*, and *x_C_* is the median of the log-normal distribution. From *x*_C_ and *w*, the arithmetic mean and the standard deviation of the population were calculated.

Magnetic characterization was carried out with a vibrating sample magnetometer from the Physical Properties Measuring System by Quantum Design (San Diego, CA, USA) in the temperature range of 5–380 K and in the magnetic field range of 0–9 T. Susceptibility measurements vs. temperature were performed under zero-field cooling (ZFC) and field cooling (FC) with μ_0_H = 2.5 mT. The relaxation measurement of the susceptibility vs. time was performed with a 2 mT field applied immediately after zero-field cooling to 5 K, keeping the sample at this temperature [4]. The aging measurement was performed by recording the magnetization vs. temperature during a cooling process from 380 to 5 K with a stop at 25 K for 3600 s, both at zero applied field and at 10 mT applied field [30]. Isothermal magnetization curves were recorded while continuously sweeping the field from +9 T to −9 T and back to +9 T. To calculate the remanent magnetization, the magnetization values were interpolated between the two data points closest to the zero field. This process was performed for both the positive and negative branches of the curve, and the average of the interpolated values was calculated to account for any asymmetry.

Similarly, for the coercive field, interpolation was applied between the data points closest to zero magnetization on both the positive and negative branches of the curve. The average values of these interpolated coercive fields provide a balanced measure of the material’s coercivity. This interpolation method ensures the accurate determination of these parameters despite the absence of exact zero-crossing points in the data.

## 3. Results and Discussion

### 3.1. Structural Characterization

**Figure 1:**
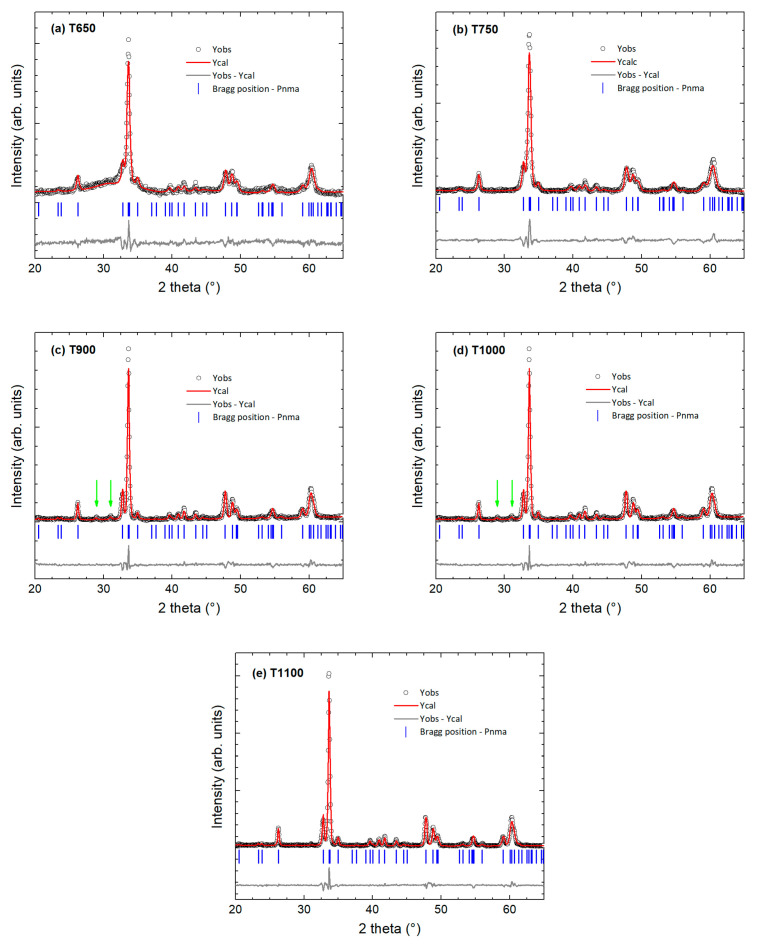
Rietveld refined X-ray powder diffraction patterns: (**a**) sample T650, (**b**) sample T750, (**c**) sample T900, (**d**) sample T1000, and (**e**) sample T1100. The experimental data (empty black dots) and the fit curve (red) are shown together with the residuals (gray bottom curve). The Bragg reflection positions of the orthorhombic Pnma main phase are represented as blue lines below the pattern. The green arrows indicate the two peaks of the secondary phase visible in the samples T900 and T1000.

Structural characterization was performed by X-ray powder diffraction (XRD); the XRD patterns of all samples are shown in Figure 1. The Rietveld refinement of the patterns was carried out using the perovskite Ho_0.5_Ca_0.5_MnO_3_ phase [22] with an orthorhombic structure (*Pnma space group 62*), similar to other half-doped lanthanide manganites [15,31]. At the lowest calcination temperature (650 °C), the perovskite phase crystallizes, reaching a diffractometric crystallite size of 39 nm (Figure 1a and Table 1). The pattern shows a small hump at low angle, compatible with residual organic compounds. With calcination at higher temperatures (from 750 °C to 1000 °C), the reflections of the Pnma phase are sharper, a signature of larger crystallites. However, a careful analysis reveals the presence of two very weak Bragg reflections at about 30°, which are attributed to a secondary phase at the limit of detectability (Figure 1b–d) identified as HoMn_2_O_5_ (orthorhombic, space group 55 *Pbam*) [32]. In these cases, a second phase was added to the refinement, without visible improvement. Considering the weak signal, the analysis of the secondary phase reveals a presence not exceeding 2–3%, within the detection limits of the experimental technique, not allowing for its further refinement. At the highest calcination temperature (1100 °C), the sample is single phase and well crystallized with a bulk-like particle size (Figure 1e and Table 1).

Table 1 presents the diffractometric crystallite sizes and strains. For non-pelletized nanopowders synthesized under our preparation conditions, the sample quality, in terms of minor local deformations, was found to decrease with crystallite size. Table 1 also reports the refined lattice parameters of the *Pnma* perovskite phase. In our case, the parameter *b* increases compared to the bulk value (7.4529 Å) with particle size reduction [15,22], while no significant difference can be observed for the *a* and *c* parameters within the experimental uncertainty.

**Figure 2:**
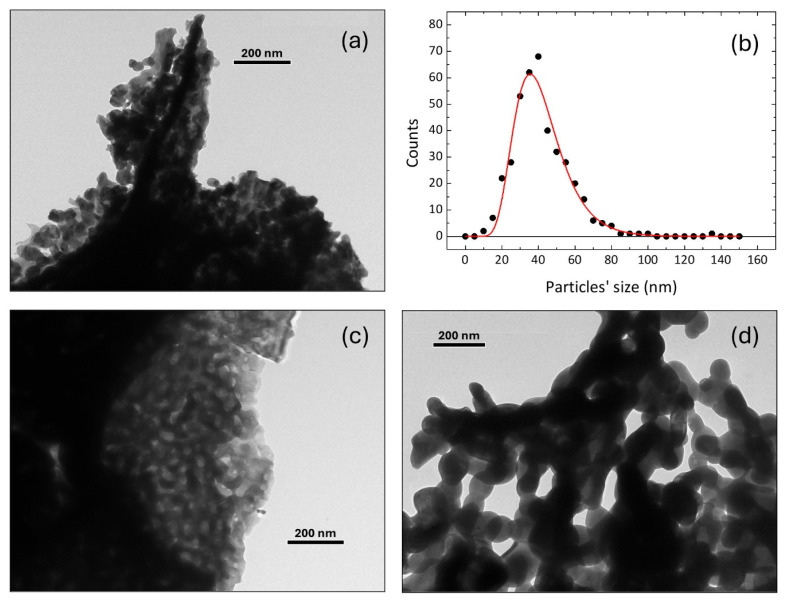
(**a**) TEM image of sample T650 with (**b**) the particle size distribution of the same sample. (**c**) Images of samples T750 and (**d**) T900.

The morphology of the particles was investigated by transmission electron microscopy. In the T650 sample, the particles are grouped into large agglomerates (Figure 2a). Particle size determination was performed on the few individual particles visible at the edges of these. The size distribution was fitted with the log-normal function (Figure 2b). The mean value of particle diameter, calculated as 42.0 (7) nm, is in very good agreement with the XRD size, supporting the presence of single crystalline nanoparticles. The standard deviation of the distribution (14.5 (8) nm) is relatively large. In the T750 sample, large agglomerates (of the order of a micrometer) are formed by the fusion of the original nanoparticles (Figure 2c). The sample shows a porous spongy texture compatible with the fusion of particles and the loss of some carbonaceous residue trapped in the original structure. In the T900 sample, the agglomerates grow to a size of several micrometers. This process destroys the previous porous structure, forming a new branched one with some hint of the original individual particles still visible (Figure 2d). The size of the particles, determined based on the very few distinguishable at the edges of the agglomerates, grows progressively (about 60 nm and 100 nm for the T750 and T900 samples). The agglomerates in the samples calcinated at higher temperatures are too large for TEM observations, showing only large ensembles. The analysis of the few grains discernable at the edges of the ensembles visible for the T1000 and T1100 samples (Figure S1) allows for a rough estimate of a grain size of about 300 nm with large standard deviation of about 100 nm. Hence, from the TEM images, one can see that the two larger samples are constituted of polycrystalline grains, since the Rietveld refinement suggests crystallites of about 106 and 135 nm in size for T1000 and T1100, respectively.

The increase in size observed for the crystallite (D) and the secondary aggregates as a function of the calcination temperature is primarily the result of enhanced atomic diffusion promoted by the increased temperature. This diffusion facilitates crystallite growth and coalescence, which occur through processes like Ostwald ripening [33] and sintering [34], where smaller crystallites dissolve or bond together to form larger ones, minimizing the overall surface energy of the system.

### 3.2. Magnetic Properties

**Figure 3:**
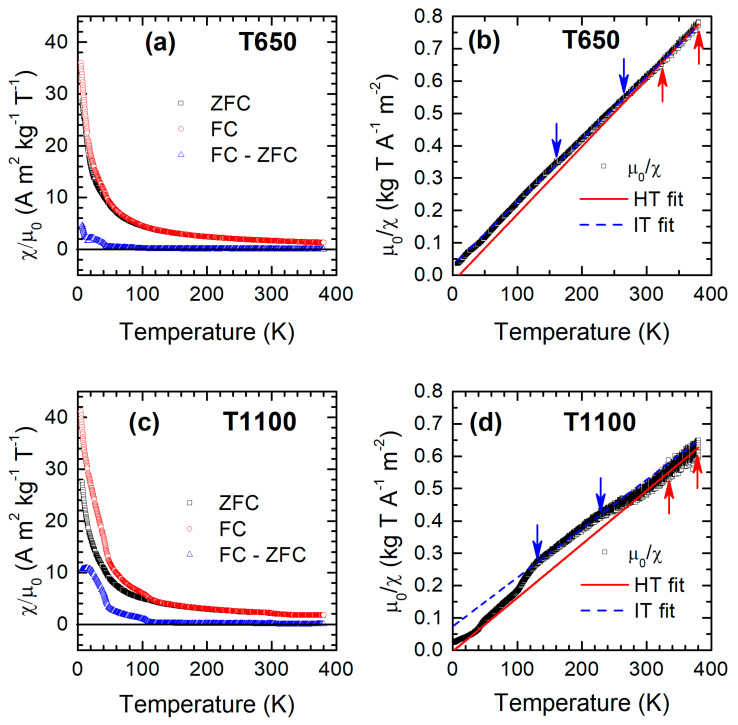
Sample T650 (**a**) ZFC susceptibility (black curve), FC susceptibility (red), and difference between them (blue). (**b**) Inverse of the ZFC susceptibility: experimental curve (black) and fit curves (red and blue). (**c**) Sample T1100 ZFC susceptibility (black curve), FC susceptibility (red), and difference between them (blue). (**d**) Inverse of the ZFC susceptibility: experimental curve (black) and fit curves (red and blue). In panels (**b**,**d**), the arrows delimit the two linear temperature ranges used in the fitting procedure.

Magnetic susceptibility (*χ*) as a function of temperature was measured for all samples in the range of 5–380 K following a standard zero-field cooling (ZFC)–field cooling (FC) protocol. The temperature dependencies of *χ*_ZFC_ and *χ*_FC_ for T650 and T1100 are shown in Figure 3a,c and Figure S2a,c,e for the other samples.

The ZFC-FC magnetization curves for the various samples share some common features. At high temperatures, all the samples are in their paramagnetic state, and no particular anomaly can be detected in the high *T* range in which the CO transition is normally observed (around 250 K for bulk Ho_0.5_Ca_0.5_MnO_3_) [13]. On cooling, all samples show two regions of thermomagnetic irreversibility, the first one at higher temperatures, starting at a slightly sample-dependent temperature *T*_irr_ of about 100–115 K, and the second one, with a much more pronounced ZFC–FC difference, setting in at a lower temperature around 40 K, which will be commented on later.

Since the analysis of the *χ*(*T*) curves does not allow us to directly establish the nature of the sequence of magnetic transitions observed, in order to obtain a clearer picture, the behavior of the inverse of the susceptibility as a function of temperature was analyzed (Figure 3b,d and Figure S2b,d,f).

For all the samples, the 1/*χ*_ZFC_ vs. *T* curve presents two linear temperature regions with different slopes, indicating that a single Curie–Weiss law [35] cannot fit the entire temperature range. Hence, we distinguish a high-temperature (HT) region and an intermediate-temperature (IT) region above and below the discontinuity. A dedicated Curie-Weiss law was fit to each region. In the present case, the Curie constant (*C*) is the sum of the contributions of Mn and Ho, and it is determined by the fit constant as *C* = *C*_Mn_ + *C*_Ho_, imposing their theoretical ratio. For all the samples, regardless of particle size, the ratio of the Curie constants of the IT and HT region is about 1.5–1.6 (Table 2).

We can compare these values with those expected by the theory of CO. Considering that Mn^3+^ and Mn^4+^ ions are present in the same quantity, the response above *T*_CO_ is given by an effective number of Bohr magnetons *p*_Mn_ = (*p*_Mn_^3+^ + *p*_Mn_^4+^)^1/2^ = 4.41. Below *T*_CO_, the pairs Mn-O-Mn + e^−^ are formed, which have a theoretical value of *p*_Mn_ = [S(S + 1)]^1/2^ = 7.94, with S = 7/2. The value of S is given by two Mn^4+^ ions with the configuration 3 d^3^ and S = 3/2 and by one electron with s = ½, which is not located on either of them. Consequently, the theoretical value of the ratio between the Curie constant of the second state and that of the first state is 1.62 [12]. The experimental values of this ratio are in agreement with the theoretical ones for all samples. These findings supports the hypothesis that the observed trend in the inverse of susceptibility originates from a charge-ordering transition, and that for Ho_0.5_Ca_0.5_MnO_3_, the charge-ordering transition occurs even at nanometric particle dimensions, unlike other nanostructured compounds of the Ln_0.5_Ca_0.5_MnO_3_ series [15,16,17,18,36]. A previous study on nanocrystalline Ho_0.5_Ca_0.5_MnO_3_ reports no direct experimental evidence of the presence of the CO transition [22]. Besides the novel synthesis method employed, which ensures high crystalline quality even for the smallest nanocrystals, preventing local variation in the structure and atomic coordination, the lack of suppression of the CO transition can be connected to the specific size of the lanthanide ions and can be explained with the use of the phase diagram in Ref. [6]. The *T*_CO_ is maximized for tolerance factors in the range of 0.889–0.905 (from Ho to Gd), while it is lower for *t* in the range of 0.908–0.924 (Sm, Pr, Nd, and La). Therefore, the bulk Ho compound intrinsically has a more stable CO state than compounds with *t* in the second range. A similar effect was recently observed after replacing La^3+^ ions with Ho^3+^ in the La_0.5_Ca_0.5_MnO_3_ system, with a significant enhancement in the T_CO_ of the original compound [37]. The previously discussed analysis of the 1/*χ*_ZFC_ vs. *T* curves permits the estimation of *T*_CO_ as the average temperature between the end of one linear region and the beginning of the other. The value for each sample is reported in Table 2.

Bulk and nanocrystalline Ho_0.5_Ca_0.5_MnO_3_ compounds exhibit a low-temperature AFM order [13,22]. In particular, neutron diffraction investigations have evidenced that AFM ordering affects only manganese and not holmium, with the occurrence of additional weak FM interactions between Mn ions [21,22]. For our samples, the Weiss constants obtained from the fitting procedure (Table 2) have positive values in the HT region and negative values in the IT one, indicating, respectively, the dominant presence of ferromagnetic and antiferromagnetic interactions in the two regions. Additionally, one can observe a reduction in the absolute value of the Weiss constant in the IT region with decreasing particle size (Table 2). This indicates a progressive weakening of the AFM interactions that, nonetheless, is not sufficient to permit the onset of the ferromagnetic order associated with the suppression of the charge-ordered state, as described in the literature for several nanostructured compounds with other lanthanide ions [15,16,17,18]. On the other hand, we are unable to identify a clear AFM transition temperature from a direct observation of the *χ*(*T*) curves. Indeed, in the lowest temperature range (LT), the trend of the susceptibility vs. *T* is predominantly hyperbolic due to the substantial paramagnetic contribution of Ho ions. If we compare the measured susceptibility with the theoretical one of Ho, we see that the paramagnetic Ho signal accounts for more than 50% of the overall signal at about 110 K, where the AFM ordering temperature is commonly observed. However, in various bulk compounds of the Ln_0.5_Ca_0.5_MnO_3_ series (Ln = Gd, Dy, Ho, and Er), the starting point of irreversibility was identified as the point at which the transition to a canted AFM state occurs [6]. Since in the *χ*(*T*) curves, a ZFC-FC irreversibility is observed in all the samples on cooling below about 110 K, we can attribute the establishment of this thermomagnetic irreversibility to a transition toward a canted AFM state and consider the irreversibility temperature *T*_irr_ as an estimate for the Néel temperature *T*_N_. The estimated values of *T*_irr_ are reported in Table 2 (see Figure 3a,c and Figure S2a,c,e).

**Figure 4:**
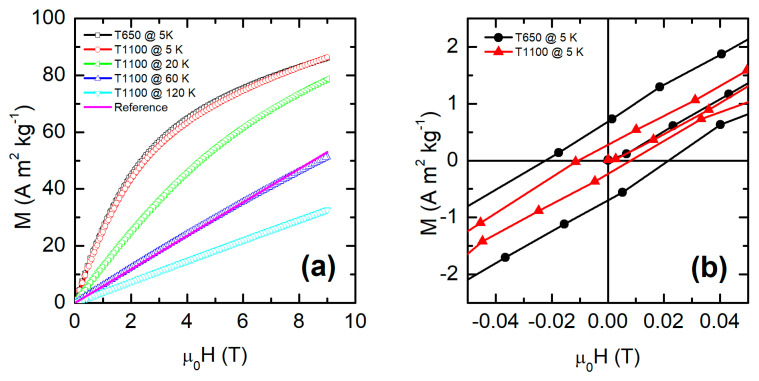
(**a**) ZFC hysteresis loop of sample T650 at 5 K (black solid curve) and of sample T1100 at 5 K (red), 20 K (green), 60 K (blue), and 120 K (cyan). As a reference, a straight line (magenta) was drawn for comparison with the 60 K curve. (**b**) ZFC hysteresis loop at 5 K of the samples T650 (black curve) and T1100 (red) in the field range from –50 to 50 mT.

To obtain more information on the magnetic behavior, in particular below the thermomagnetic irreversibility temperature, the ZFC magnetization versus field curves *M*(*H*) were measured at different temperatures. Figure S3 shows the *M*(*H*) curves measured at 5 K for all the samples, while Figure 4a shows the curves recorded at 5 K for the extreme samples of the series, i.e., T650 and T1100, and the evolution of the *M*(*H*) response with a temperature of T1100.

In T1100, a paramagnetic linear response is observed at temperatures higher than 120 K, while a deviation from linearity can be seen starting from 60 K. At lower temperatures, hysteretic behavior is observed. At 5 K, the *M*(*H*) curve exhibits larger hysteresis, with a larger remanence magnetization (*M*_R_) and coercive field (*H*_C_) with decreasing particle size, as visible in Figure 4b and Figure S3b and from the values of *M*_R_ and *H*_c_ reported in Table 3. The same trend is shown for magnetization at 9 T. Sample T650 does not follow this trend, likely due to the presence of some residual organic material, as discussed above. The increase in these parameters with decreasing particle size is related to the contribution of the surface spin canting (associated with the reconstruction of the magnetic surface), which grows with decreasing size [14,18,38]. This canting grows due to the weakening of AFM interactions, discussed above, and the larger surface-to-volume ratio. Another feature of the 5 K *M*(*H*) curves is that the magnetization at 9 T is lower than the theoretical value for paramagnetic Ho; the Brillouin function (B_J_(x) = 0.963) yields a magnetization of 131 A m^2^ kg^−1^. This reduced magnetization confirms the presence of interactions between the magnetic moments of Ho and Mn, as observed by neutron diffraction [22].

**Figure 5:**
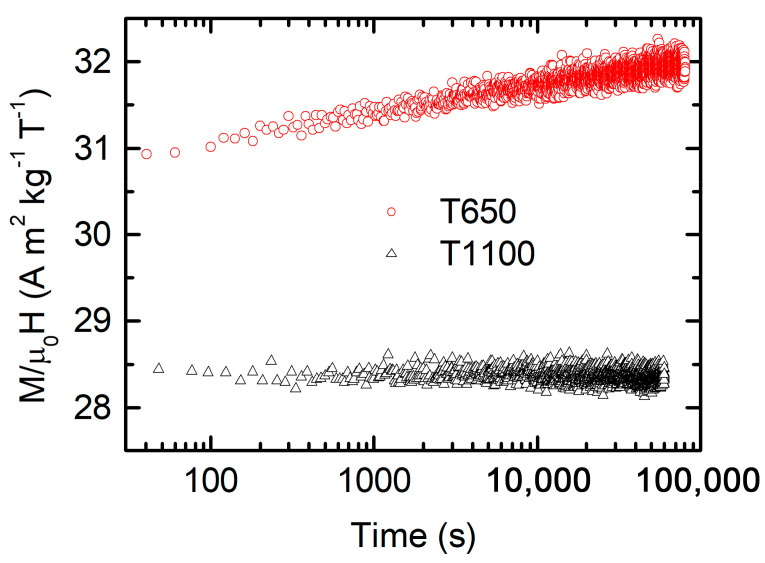
Susceptibility vs. time at 5 K and 2 mT of sample T650 (red empty dots) and sample T1100 (black empty dots). The field was applied immediately after a zero-field cooling down to 5 K.

The magnetic behavior of the samples in the lowest-temperature region appears rather complex, as can be seen from the trend of the *χ*(*T*) curves, showing thermomagnetic irreversibility around 40 K and a peaked feature of the ZFC-FC difference, particularly evident in the intermediate samples of the series (T750, T900, T1000, Figure S2a,c,e). The presence of glass-like states in this temperature region is reported in the literature [4,6,14]. To verify this hypothesis, AC susceptibility measurements were performed in all samples. However, they do not provide significant information because, similarly to the DC measurements, the AC curves are dominated by the paramagnetic Ho susceptibility, as also reported in Ref. [4]. Therefore, to investigate possible glassy dynamics, we performed relaxation measurements recording susceptibility vs. time for the extreme samples in the series, T650 and T1100. (Figure 5). No time dependence of susceptibility was observed for T1100, while is a slight but evident time dependence was found for T650, which is one of the typical effects of magnetic frustration [4] or FM-AFM ordering, observed also in perovskites with an antiferromagnetic ground state [39].

It is worth mentioning that, besides the relaxation curves, we tested aging effects by recording a cooling process from 380 to 5 K with a stop at 25 K for 3600 s at zero applied field and at 10 mT applied field [30] (Figures S4 and S5). Interestingly, neither sample showed any aging effect in the cooling curve, even though the stop at 25 K should be well below the typical occurrence of a glassy state (45 K) in the compounds of the series [5,14,17,40]. In light of the whole investigation of the samples, one can conclude that they do not exhibit aging effects, thus excluding the presence of a canonical spin-glass state [4,41] with a glassy temperature about 45 K, as often suggested in the literature. This is in line with the recent observations of La_0.5-x_Ho_x_Ca_0.5_MnO_3_, where even a small Ho doping enhances CO and suppresses the spin-glass-like state observed in the original La_0.5_Ca_0.5_MnO_3_ compound [42] in favor of canted AFM one, as suggested for bulk Ho_0.5_Ca_0.5_MnO_3_ [6].

## 4. Conclusions

In this work, we investigated the magnetic properties of a set of nanostructured samples of Ho_0.5_Ca_0.5_MnO_3_, chosen as a model system for the Ln_0.5_Ca_0.5_MnO_3_ series of manganites with a perovskite structure featuring small lanthanide (Ln) ions partially substituted by Ca ions. The aim was to explore the potential effects of reducing the material’s dimensionality at the nanoscale. The samples were prepared from a single batch using a modified sol–gel approach, followed by the calcination of the original powder at different temperatures. XRD and TEM analysis confirmed that this hybrid approach promotes high crystalline quality, ensuring the same chemical composition for all samples, and allows for the production of samples with different average nanocrystal sizes, modulated by the calcination temperature used for each one in the range of 39–135 nm. DC magnetization vs. temperature measurements indicated that the charge-ordering transition is not suppressed, even in the sample with the smallest crystalline grains, contrary to what has been observed in studied compounds of the series with La, Nd, Pr, and Sm, usually attributed to nanoscale induced disorder effects. Here, we observe that the charge-ordering transition can be observed even at the nanoscale for Ho_0.5_Ca_0.5_MnO_3_. We connect this observation to the preserved crystalline structure and the small size of Ho^3+^ ions, resulting in a small tolerance factor compared to other compounds in the Ln_0.5_Ca_0.5_MnO_3_ series, which promotes the stabilization of the CO phenomenon. Additionally, this research explored the presence of glassy phenomena, commonly seen in this class of nanostructured materials. In order to enhance our understating beyond simplistic qualitative observations, we performed a typical comprehensive characterization of the spin-glass system using AC susceptibility, relaxation, and aging measurements. The results confirm mixed behavior. While no frequency dependence of AC susceptibility vs. temperature could be identified, mostly due to the paramagnetic-like background produced by Ho ions, only the sample with the smallest particles manifested slow relaxation dynamics. Furthermore, none of the samples manifested aging effects compatible with a glassy ordering temperature below about 45 K, often suggested for other compounds in the series. In conclusion, despite the presence of complex dynamics typical of glassy phenomena, the Ho_0.5_Ca_0.5_MnO_3_ compound cannot be intrinsically classified as a canonical spin glass, regardless of the size of its nanocrystals.

In summary, this work not only provides significant insights into the charge-ordering and glassy dynamics of nanostructured Ho_0.5_Ca_0.5_MnO_3_ but also establishes a robust general framework for studying nanostructured manganites, the multifunctional properties of which hold great potential for technological applications in advanced magnetic and electronic device, at low and room temperatures, such as magnetic and temperature sensors, magnetic refrigerators, spintronic devices, and protectors against electromagnetic pulses, just to name a few [43,44,45,46,47,48].

## Figures and Tables

**Table 1:** Diffractometric crystallite size (*D*), orthorhombic lattice parameters (*a*, *b*, *c*), strain (*ε*), reliability index parameters *R_wp_* (weighted residual error) and *R_B_* (Bragg factor), and goodness of the fit *Gof = R_wp_/R_exp,_ = χ*. Standard errors given in parentheses.

Sample	*D* (nm)	*a* (Å)	*b* (Å)	*c* (Å)	*ε* (degrees)	*R_wp_* (%)	*R_exp_* (%)	*R_B_* (%)	*Gof*
T650	39 (3)	5.458 (1)	7.470 (2)	5.301 (1)	0.0029 (2)	10.58	6.70	8.46	1.52
T750	53 (2)	5.459 (2)	7.467 (2)	5.298 (1)	0.0038 (4)	12.49	7.52	9.99	1.66
T900	86 (3)	5.462 (1)	7.460 (1)	5.310 (1)	0.0027 (1)	12.69	8.19	10.03	1.55
T1000	106 (3)	5.462 (1)	7.460 (1)	5.310 (1)	0.0025 (1)	12.71	8.26	10.06	1.54
T1100	135 (5)	5.459 (1)	7.454 (1)	5.306 (1)	0.0023 (1)	11.08	7.40	8.66	1.50

**Table 2:** Charge-ordering temperature (*T*_CO_), starting point of irreversibility (*T*_irr_) indicative of a canted AFM ordering, Weiss constants at HT (*θ_HT_*) and IT (*θ_IT_*), and ratio of the IT and HT Curie constants of Mn. Errors are shown in parentheses.

Sample	*T*_CO_ (K)	*T*_irr_ (K)	*θ_HT_* (K)	*θ_IT_* (K)	*C*_Mn IT_/*C*_Mn HT_
T650	292 (10)	102 (5)	10 (3)	−16 (2)	1.50 (12)
T750	270 (10)	108 (5)	15 (6)	−23 (1)	1.49 (12)
T900	278 (10)	111 (5)	21 (4)	−30 (3)	1.59 (13)
T1000	274 (10)	115 (5)	9 (3)	−39 (4)	1.50 (12)
T1100	293 (30)	115 (5)	2 (3)	−46 (5)	1.53 (12)

**Table 3:** Parameters extracted from *M*(*H*) curves at 5 K: remanence magnetization (*M*_R_), coercive field (*µ_0_H*_C_), and magnetization at 9 T (*M*_9T_). Errors are given in parentheses.

Sample	*M*_R_ (A m^2^ kg^−1^)	*µ_0_H*_C_ (mT)	*M*_9T_ (A m^2^ kg^−1^)
T650	0.66 (3)	22 (2)	86.0 (1)
T750	0.93 (3)	36 (2)	95.3 (1)
T900	0.59 (1)	22.3 (5)	91.0 (1)
T1000	0.33 (1)	11.4 (2)	85.6 (1)
T1100	0.25 (2)	9 (1)	86.3 (1)

## Data Availability

The raw data supporting the conclusions of this article will be made available by the authors on request.

## Supplementary Materials

The following supporting information can be downloaded at: https://www.mdpi.com/article/10.3390/nano15030203/s1, Figure S1: TEM images of sample T1000 and T1100; Figure S2: ZFC-FC curves and relative inverse of susceptibility of samples T750, T900, and T1000; Figure S3: ZFC magnetization vs. field curves at 5 K for all the samples; Figure S4: Aging measurements at 25 K for sample T650; Figure S5: Aging measurements at 25 K for sample T1100.
