# Supplementary material for: Charge-Ordering and Magnetic Transitions in Nanocrystalline Half-Doped Rare Earth Manganite Ho0.5Ca0.5MnO3
Source: Nanomaterials (Basel). 2025 Jan 27;15(3):203. doi: 10.3390/nano15030203 (PMC11820790; doi:10.3390/nano15030203)
Supplement: Supplementary file 1 [file nanomaterials-15-00203-s001.zip › nanomaterials-3372004-supplementary.pdf]

Article

Charge-Ordering and Magnetic Transitions in Nanocrystalline Half-Doped Rare Earth Manganite $Ho_{0.5}Ca_{0.5}MnO_3$

Giuseppe Muscas, Francesco Congiu *, Alessandra Geddo Lehmann and Giorgio Concas

Dipartimento di Fisica, Università di Cagliari, I-09042 Monserrato, CA, Italy; g.muscas@unica.it (G.M.); a.geddolehmann@gmail.com (A.G.L.); gconcas@unica.it (G.C.)

* Correspondence: franco.congiu@dsf.unica.it

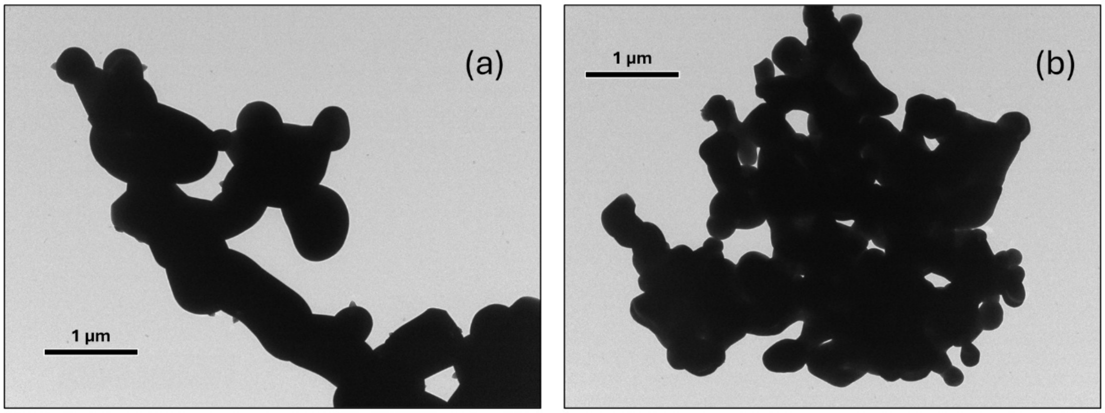

Figure S1: TEM image of the typical large agglomerates constituting sample T1000 (a) and sample T1100 (b).

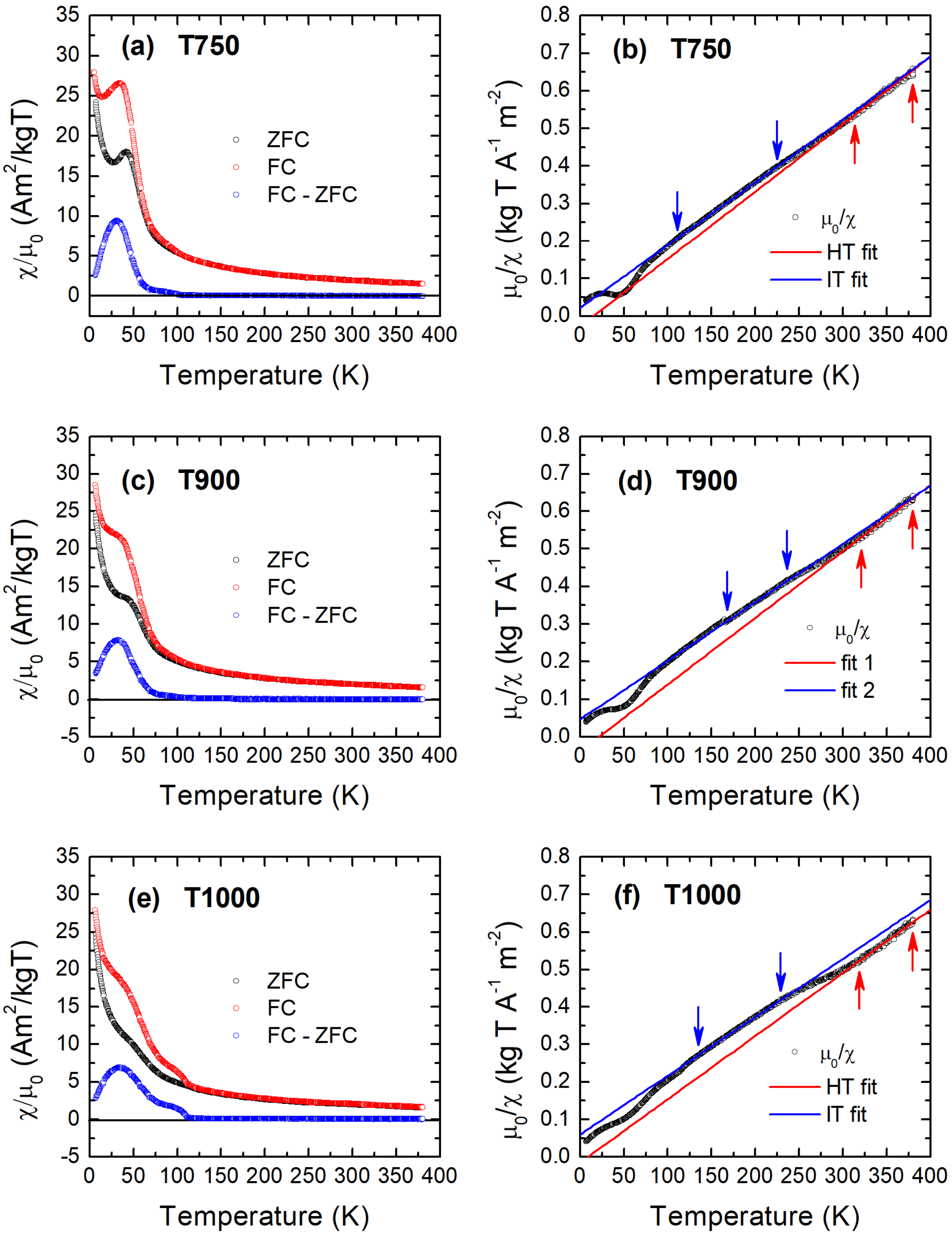

Figure S2: Sample 750: (a) ZFC susceptibility (black curve), FC susceptibility (red) and difference between them (blue) of the sample T750. (b) Reciprocal ZFC susceptibility; the experimental data (black curve) and the linear fit curves (red) are shown. (c) and (d): the same for the sample T900. (e) and (f); the same for the sample T1000. In panels (b), (d) and (f) the arrows delimit the two linear temperature ranges used in the fitting procedure.

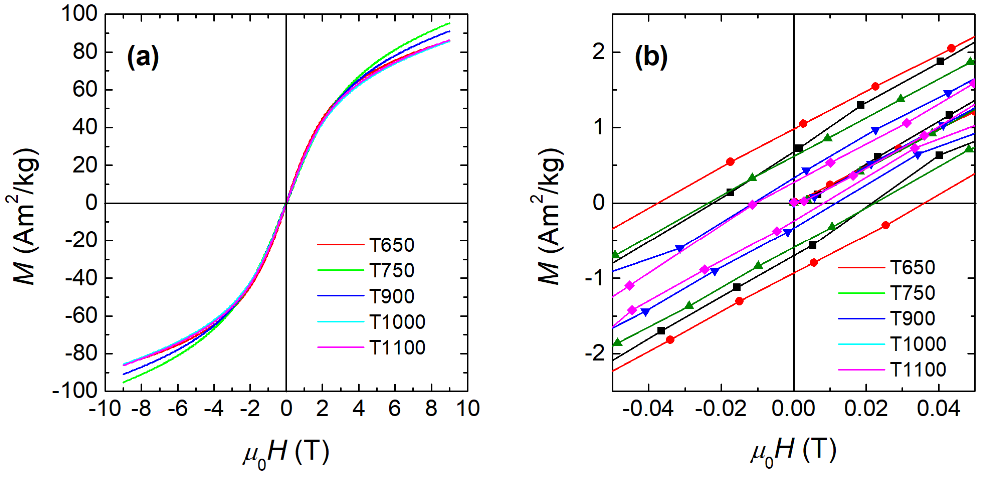

Figure S3: (a) ZFC magnetization vs field curves at 5 K of the samples T650 (red curve), T750 (green curve), T900 (blue curve), T1000 (cyan curve), and T1100 (magenta curve). (b) Same as (a) in the field range from –50 to 50 mT.

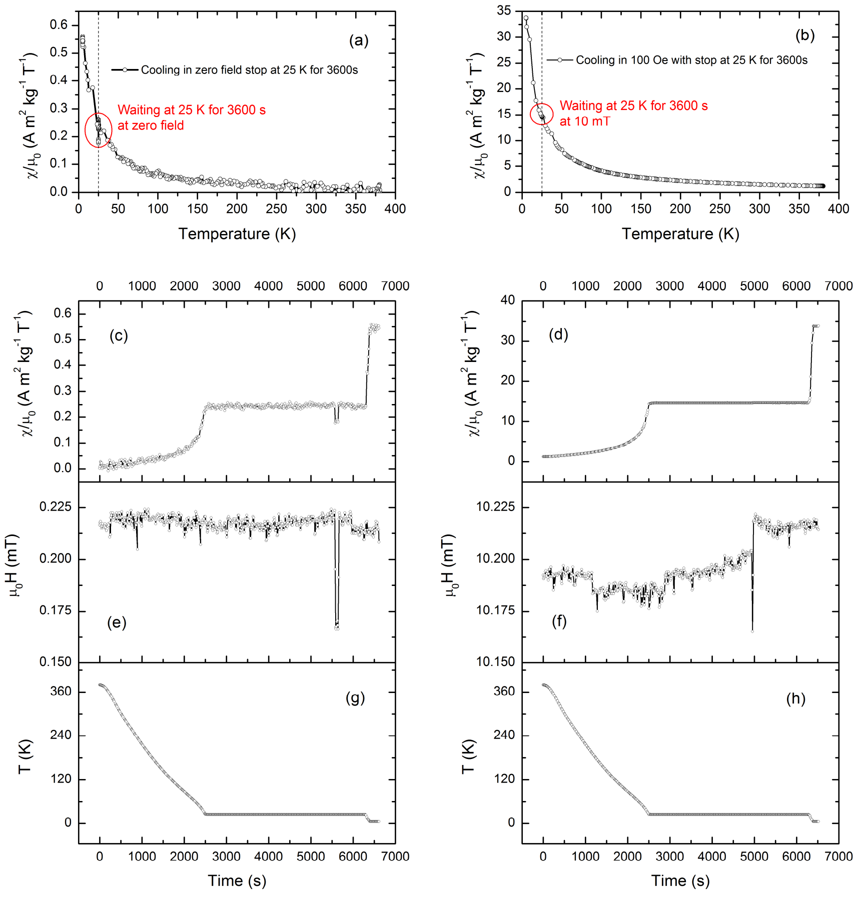

Figure S4: Aging measurements at 25 K for sample T650 in zero field (a) and in 10 mT field cooling protocol (b). Time evolution of susceptibility (c), residual magnetic field (e), and temperature (g) in zero-field cooling protocol. Time evolution of susceptibility (d), applied magnetic field (f), and temperature (h) in 10 mT field cooling protocol.

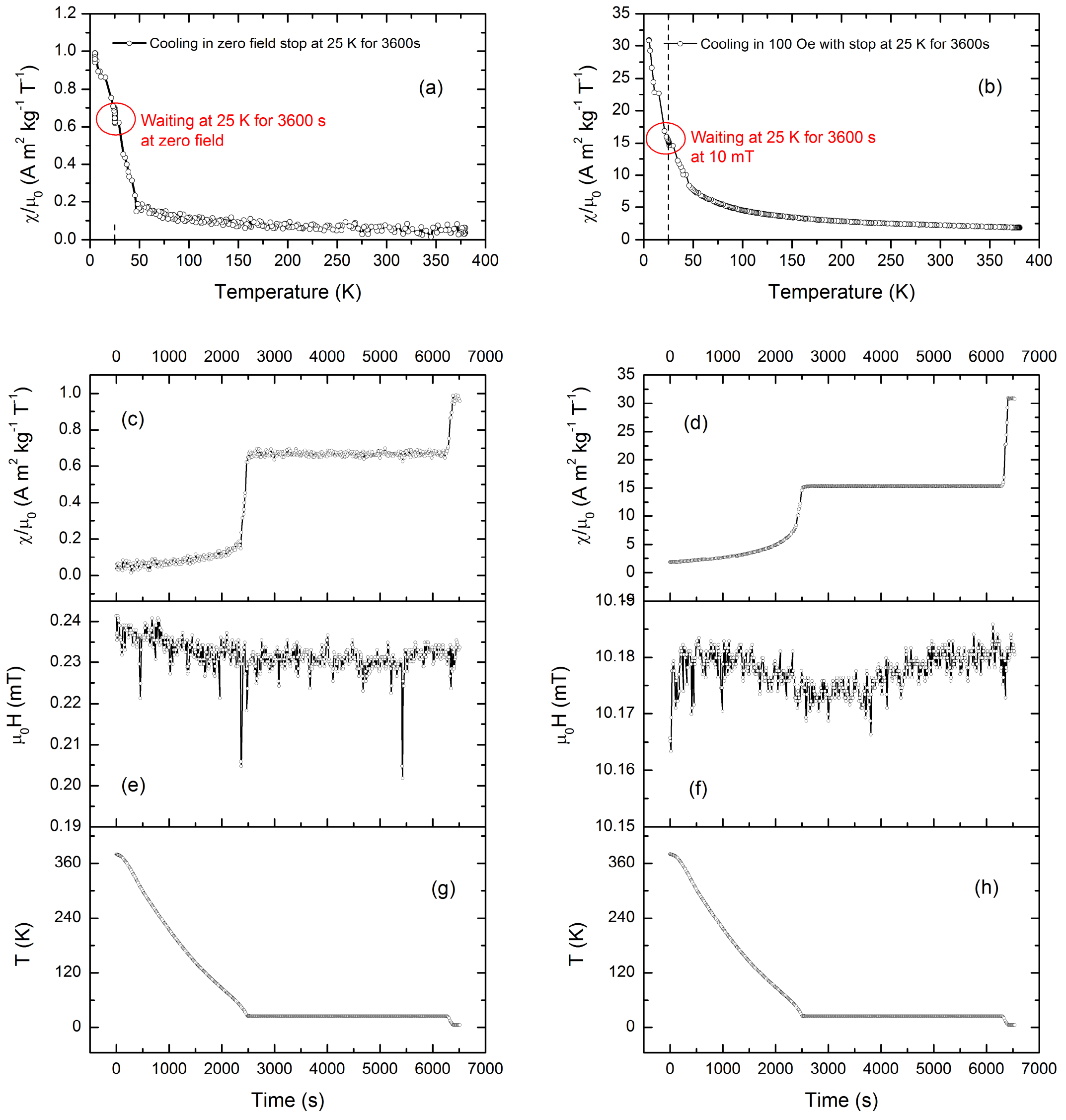

Figure S5: Aging measurements at 25 K for sample T1100 in zero field (a) and 10 mT field cooling protocol (b). Time evolution of susceptibility (c), residual magnetic field (e), and temperature (g) in zero-field cooling protocol. Time evolution of susceptibility (d), applied magnetic field (f), and temperature (h) in 10 mT field cooling protocol.
